# Millisecond‐scale behaviours of plankton quantified in vitro and in situ using the Event‐based Vision Sensor

**DOI:** 10.1002/ece3.70150

**Published:** 2024-08-27

**Authors:** Susumu Takatsuka, Norio Miyamoto, Hidehito Sato, Yoshiaki Morino, Yoshihisa Kurita, Akinori Yabuki, Chong Chen, Shinsuke Kawagucci

**Affiliations:** ^1^ Sony Group Corporation Minato‐ku Japan; ^2^ Super‐Cutting‐Edge Grand and Advanced Research (SUGAR) Program, Institute for Extra‐Cutting‐Edge Science and Technology Avant‐Garde Research (X‐STAR) Japan Agency for Marine‐Earth Science and Technology (JAMSTEC) Yokosuka Kanagawa Japan; ^3^ Institute of Life and Environmental Sciences University of Tsukuba Tsukuba Ibaraki Japan; ^4^ Fishery Research Laboratory Kyushu University Fukutsu Fukuoka Japan; ^5^ Marine Biodiversity and Environmental Assessment Research Center (BioEnv), Research Institute for Global Change (RIGC) Japan Agency for Marine‐Earth Science and Technology (JAMSTEC) Yokosuka Kanagawa Japan

**Keywords:** computer vision, deep sea, dynamic vision sensor, event camera, high‐speed camera, marine particles

## Abstract

The Event‐based Vision Sensor (EVS) is a bio‐inspired sensor that captures detailed motions of objects, aiming to become the ‘eyes’ of machines like self‐driving cars. Compared to conventional frame‐based image sensors, the EVS has an extremely fast motion capture equivalent to 10,000‐fps even with standard optical settings, plus high dynamic ranges for brightness and also lower consumption of memory and energy. Here, we developed 22 characteristic features for analysing the motions of aquatic particles from the EVS raw data and tested the applicability of the EVS in analysing plankton behaviour. Laboratory cultures of six species of zooplankton and phytoplankton were observed, confirming species‐specific motion periodicities up to 41 Hz. We applied machine learning to automatically classify particles into four categories of zooplankton and passive particles, achieving an accuracy up to 86%. At the in situ deployment of the EVS at the bottom of Lake Biwa, several particles exhibiting distinct cumulative trajectory with periodicities in their motion (up to 16 Hz) were identified, suggesting that they were living organisms with rhythmic behaviour. We also used the EVS in the deep sea, observing particles with active motion and periodicities over 40 Hz. Our application of the EVS, especially focusing on its millisecond‐scale temporal resolution and wide dynamic range, provides a new avenue to investigate organismal behaviour characterised by rapid and periodical motions. The EVS will likely be applicable in the near future for the automated monitoring of plankton behaviour by edge computing on autonomous floats, as well as quantifying rapid cellular‐level activities under microscopy.

## Graphical abstract

Marine particles, including plankton and particulate organic matter, are fundamental elements within marine ecosystems, and understanding their dynamics will enhance our knowledge of marine ecology. This study developed a new method to quantitatively evaluate the dynamics of marine particles using the recently developed Event‐based Vision Sensor. Despite its low power and memory consumption, this method is characterised by high temporal resolution and sensitivity.

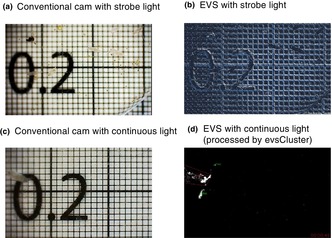

## 1 INTRODUCTION

Visual observation is a fundamental piece of information for biology. Human beings have been documenting the visual information of species, such as their general appearance and behaviour, from the early history as is evident from cave art and murals (Brumm et al., 2021). Recording of organismal behaviour and morphology went from drawings to photography and, in the last century, video cameras were developed. Videos allow us to objectively capture organisms' behaviour, and opened avenues of research into the functions and motions of individual bodies and body parts, spatiotemporal tracking of individuals in the natural environment, and physical‐social interactions (e.g., grazing, mating, etc.) among individuals and species. In addition to qualitative descriptions based on video recordings, quantitative data extracted by mathematical analyses enabled us to objectively interpret and understand the behaviour of species. Typically, videos are frame‐based, meaning they are a series of still images where information from all pixels (i.e. individual sensors corresponding to a pixel) on the image sensor is recorded synchronously as a ‘frame’.

High‐speed video imaging is a technology allowing the recording of many more frames per unit time than what the human eye can normally perceive. While human vision image acquisition is no faster than the order of 100 frames per second (fps) (Potter et al., 2014), high‐speed videos are capable of >1000 fps (one frame per millisecond) (e.g., Thoroddsen et al., 2008) and have revolutionised visual observations of biological phenomena. For example, high‐speed video analyses revealed quantitative data and trends in the behaviour of planktons, such as changes in speed/acceleration/orientation at the millisecond‐scale (Buskey et al., 2002). However, the current high‐speed video imaging techniques are still frame‐based, and since shorter exposure time leads to less light per frame per photosite sensor, there is a demand for strong lighting to achieve imaging at a millisecond timeframe. This also means the system consumes a high amount of power and can only be filmed over a short duration due to heavy memory consumption (Table 1). This strong lighting required for frame‐based high‐speed imaging can also influence animal behaviour and impact the results, as even typical working light from a ship causes behaviour changes in fish and zooplankton at night due to phototaxis (Berge et al., 2020). These commercially available high‐speed video systems with lighting setups are typically cumbersome and costly, limiting their use in natural ecosystem monitoring.

**TABLE 1:** A comparison table with GoPro HERO8, FASTCAM Nova S6 and EVS prototype.

	GoPro HERO8	FASTCAM Nova S6	EVS prototype
Resolution	1280 × 820	1024 × 768	1280 × 720
Frame rate	240 fps	9000 fps	10,000 fps
			Equivalent temporal precision
Data size per minute	340 MB (MP4)	594 GB	200–500 MB
Power consumption with memory and peripheral circuits	1.2–1.5 W	138 W^a^	10.2–12.5 W^b^
Light power consumption	33 W^c^	120 W^d^	1 W

^a^ https://www.webj.co.jp/wj/wp‐content/uploads/2019/01/7aec9ebad50b2718d72a0c3b411b6234.pdf

^b^ EVS prototype camera including FPGA and M.2 memory.

^c^ *Edokko Mark 1 model COEDO* light power consumption.

^d^ https://www.h‐repic.co.jp/products/fiber/fiber_led/la‐hdf_8010

**FIGURE 1:**
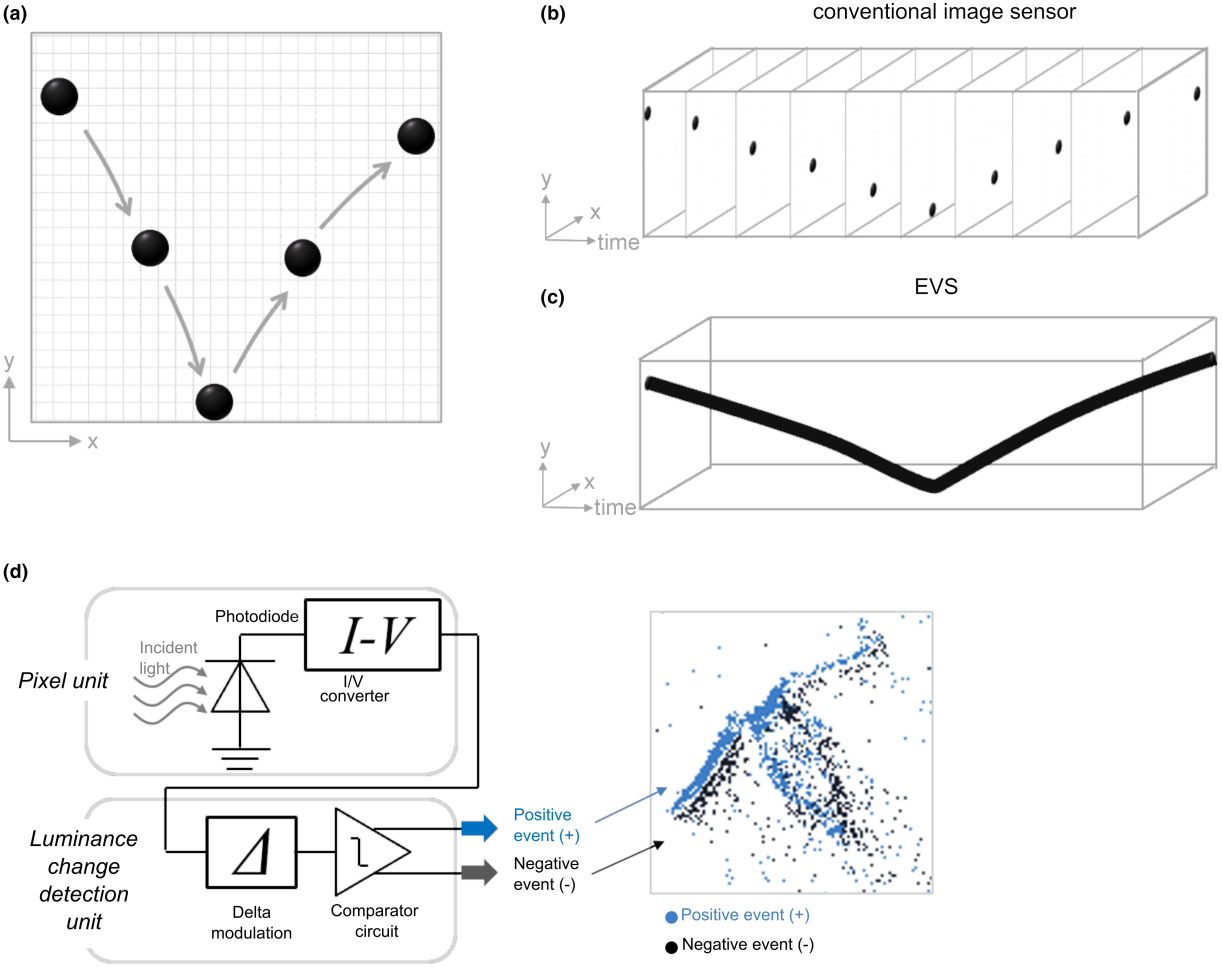
General concept of the Event‐based Vision Sensor (EVS). (a–c) Differences between a conventional frame‐based image sensor and the EVS when recording a bouncing ball. (b) Frame‐based image sensors record all pixel information together, in ‘frames’. (c) The EVS detects brightness changes asynchronously at each pixel and only those pixels with changes are recorded. (d) The mechanism of an EVS as a circuit diagram and output image.

The Event‐based Vision Sensor (EVS) (Gallego et al., 2022; Lichtsteiner et al., 2008), also known as the event camera, dynamic vision sensor, neuromorphic camera, or silicon retina, is a bio‐inspired sensor. The EVS is designed to emulate how the human eye senses light. The human eye functions in such a way that the receptors on the retina convert light into visual signals to be sent to the brain. Subsequently, neurons identify light and shade conditions, which are then relayed to the visual cortex in the brain via retinal ganglion cells. By mimicking this process, the EVS differs from frame‐based systems in that the information in each pixel is recorded separately, when there is a change in brightness (Figure 1a–c). Each pixel records a different output depending on the polarity of the brightness change. For example, an increase in brightness is recorded as a positive (+) change, while a decrease in brightness is recorded as a negative (−) change (Figure 1d). These changes are called *events*, hence the name EVS. The EVS generates a dataset consisting of coordinates (*X*/*Y*), polarities (+/−), and timestamps of *events* in each pixel – meaning there is no need to record information from those pixels with no changes over time. This allows a much reduced data size as well as memory and power consumption, meaning it can track the movement of objects much more efficiently under low light conditions while having less power and memory consumption when compared to conventional frame‐based video systems such as GoPro HERO8 and the high‐speed FASTCAM Nova S6 (Table 1). The resulting four‐dimensional dataset can be directly used to analyse the motion of objects, but the same data can also be used to visually simulate the recorded changes for cross‐checking the object's tracks with human eyes (Figure 1d). The file size is two or more magnitudes smaller, while the power consumption is an order of magnitude less than frame‐based high‐speed video cameras (e.g. FASTcam Nova S6) currently on the market. Since the commercial distribution of the EVS began in 2008 (Lichtsteiner et al., 2008), it has been anticipated as a solution to improved computer vision with applicability to devices such as self‐driving cars (Maqueda et al., 2018). These attributes of the EVS, however, mean that it also has great applicability to observe and analyse the detailed movement and behavioural patterns of living organisms.

Here, we report the first application of the EVS to observing and analysing the behaviour of particles, including living plankton and passive particles, in the aquatic environment. Previously, only passive particle motions (Ren et al., 2022; Wang et al., 2020; Zhang et al., 2023) and terrestrial animal behaviours (Hamann et al., 2024) have been analysed by the EVS. We developed a piece of software named ‘evsCluster’ to analyse the EVS data. Living planktons are representative ‘active particles’ in natural aquatic systems, and in situ behaviours of the living organisms and their ecological functions are central issues of aquatic biology as well as biological limnology and oceanography (e.g. McManus & Woodson, 2012). At the same time, numerous ‘inactive particles’ such as dead organisms, faecal pellets, minerals, and their aggregates float in a matrix of liquid flow and are gravitated toward the bottom. Our EVS system, protected by water‐proof (and pressure‐tolerant for deep water operations) housings, successfully identified in situ swimming behaviours of planktons even in dense particle storms and marine snow at the bottoms of both a lake and an ocean. Testing the system mounted onto a microscope in a controlled laboratory aquarium setting shows that the system can automatically distinguish three zooplankton species among mixed suspended particles. Our results demonstrate a strong potential to apply the EVS to life science research, beyond industrial applications.

## 2 METHODS

### 2.1 Hardware and experimental settings

#### 2.1.1 Sensor and camera systems

We used a stacked Event‐based Vision Sensor (IMX636, manufactured by Sony Semiconductor Solutions Corporation) with a resolution of 1280 × 720 pixels and 4.86 × 4.86 μm individual pixel size. In this study, the same model of EVS sensor was fitted on two different camera systems to suit in vitro and in situ observations. For in vitro observation, we used a prototype EVS camera system, a developer's model that was controlled and powered via USB‐C using a Windows laptop. For in situ observation in lacustrine and deep‐sea environments, we modified the prototype EVS camera system to prepare a stand‐alone EVS camera system that did not need to be connected to a laptop. This stand‐alone EVS camera system consisted of the EVS (IMX636, Sony), a field‐programmable gate array (FPGA) (XCZU3EG‐2SFVC784, Xilinx), an Arduino board (Arduino Pro Mini 3.3 V 8 MHz, Aideepen), an M.2 memory card (SB‐RKTQ‐8 TB, SABRENT), and Bluetooth peripheral circuits (sh‐hc‐06, DSD TECH). This in situ EVS observation system was operated by an external power supply through the USB Power Delivery standard and a 25,600‐mAh portable battery that allowed >6 h of continuous operation. The optical interface of both camera systems was developed for the CS‐mount lens standard.

We further developed dual‐vision systems, which combines two imaging systems – an EVS and a conventional frame‐based camera – for simultaneous monitoring, in order to obtain complementary images for size and shape recognition between an EVS camera and a conventional frame‐based camera for the evaluation of EVS performance. For in vitro laboratory work, both the EVS system and a conventional camera (HOZAN USB Camera L‐835) were mounted on a stereomicroscope (YS05T, Micronet Inc.) and the optical axis from the same objective lens was split to take complementary videos in both systems. For in situ observations in natural aquatic environments, a cubic beam splitter (ELIOTEC CORP.) was placed behind the objective lens to provide the same images to both the EVS system and the conventional camera (GoPro HERO8 or HERO7, modified to C‐mount, GoPro Inc., Figure S1).

**FIGURE 2:**
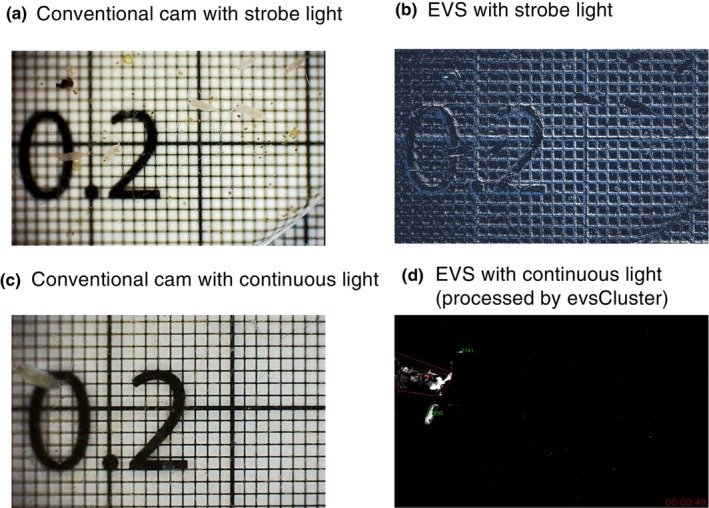
Comparison between conventional and Event‐based Vision Sensor (EVS) cameras. (a) Light micrograph taken using the conventional camera of the dual‐vision system with strobe light, on 0.2 mm square gridded graphing paper. Both living copepods and background grids are captured. (b) An image recorded by EVS simultaneously with (a). (c) Light micrographs taken using a conventional camera with continuous light. (d) An image recorded by EVS simultaneously with (c), in which only moving objects are captured. Frames and numbers (red and green) are generated through evsCluster processing (see Figure 4).

To demonstate and compare characteristics of images recorded by the EVS and conventional cameras, examples recorded by the dual‐vision system under strobe and continuous light conditions are shown in Figure 2. As the EVS captures the change in brightness for each pixel, recording with strobe light allows the EVS to capture the outline of static objects, generating comparable images between the two camera systems (Figure 2a,b). Such EVS recordings with strobe light on a scale (like a gridded graph paper) allowed us to calibrate the pixel size of that specific recording in conventional units (such as millimetres). Conversely, images taken by the two camera systems under continuous light are distinct. While both static and moving objects are captured by the conventional camera (Figure 2c), only moving objects are captured by the EVS (Figure 2d). Comparisons under well‐lit conditions show that body lengths captured by the EVS are quantitatively consistent with those measured from the conventional frame‐based camera.

#### 2.1.2 In vitro evaluation

For the in vitro evaluation of the EVS sensor, we used four species of zooplankton and two species of phytoplankton and observed them with an EVS system in the laboratory (Table 2). The paracalanid marine copepod *Parvocalanus crassirostris* was purchased from Pacific Trading Co., Ltd. and reared in a 10 L tank. Eggs of the brine shrimp *Artemia* sp. were purchased from Kyorin Co., Ltd., and hatched by aeration in seawater. Sexually mature individuals of the true limpet *Nipponacmea fuscoviridis* were collected from Hiraiso, Ibaraki Prefecture, and Tsuyazaki, Fukuoka Prefecture in Japan; mature individuals of the sea star *Patiria pectinifera* were collected from Tsuyazaki and Asamushi, Aomori Prefecture in Japan. These two species were reared in aquaria and in vitro fertilisation was used to obtain their larvae, as previously described (Hashimoto et al., 2012; Koga et al., 2010). Two phytoplankton species, *Chattonella marina* (NIES‐1) and *Heterosigma akashiwo* (NIES‐5), were provided by the National Institute for Environmental Studies (NIES). through the National BioResource Project (NBRP) of the Ministry of Education, Culture, Sports, Science and Technology (MEXT), Japan. During observation, the stereomicroscope was configured for horizontal viewing because the target movements were vertical movements of swimming planktons as well as particles settling under the influence of gravity (Figure S2A,B). A narrow acrylic aquarium with an inner thickness of 5 mm between the walls was placed in front of the object lens for observation (Figure S2C). Several dozen individuals of the target organism were introduced into the aquarium, and the movement of particles was recorded. The behaviour of each plankton species was observed by the EVS and the output analysed. Three zooplankton species, including *Parvocalanus crassirostris*, *N. fuscoviridis*, and *Patiria pectinifera*, were recorded together in a random mixture to test the capacity of the computer software to tease these species apart.

**TABLE 2:** Organisms examined in this study.

Phylum	Species	Developmental stage(s)
Arthropoda	*Parvocalanus crassirostris*	Nauplius larva, copepodite, adult
Arthropoda	*Artemia* sp.	Metanauplius larva
Mollusca	*Nipponacmea fuscoviridis*	Veliger larva
Echinodermata	*Patiria pectinifera*	Bipinnaria larva
Ochrophyta	*Chattonella marina*	–
Ochrophyta	*Heterosigma akashiwo*	–

#### 2.1.3 In situ evaluations

In order to evaluate the applicability of the EVS system to observing planktonic organisms in natural aquatic systems, we attempted to record and analyse the behaviour of particles in Lake Biwa and Suruga Bay, Japan. For in situ observation at Lake Biwa, the EVS camera system with a Variable Focus Iris Lens (C‐Mount, Focal length: 4–12 mm, Aperture setting: F16–F22) was contained in a glass sphere mounted on the stand‐alone monitoring system *Edokko Mark 1* model COEDO (Kawagucci et al., 2020) (see section 4.1, Figure S3). The COEDO provides white light for conventional frame‐based video imaging. The EVS system mounted on the COEDO (Figure S3) was deployed at the bottom of Lake Biwa at a depth of 60 m on March 8, 2022 (Figure S4, Yamada et al., 2021). The main body of the COEDO was buoyant from the glass spheres, and so was anchored by a ballast and rope during the deployment. The altitude of the EVS camera when deployed was ca. 1 m from the lake floor. The water temperature in situ was approximately 6.8°C–8.5°C. The recording was conducted during the daytime from 11:36 to 20:22 (8 h 46 m) Japan standard time (JST) under sunlight with additional lighting from the COEDO's main body. The lake water appeared cloudy at the surface, due to turbid inflows from neighbouring rivers due to heavy snowfalls several days prior to the observation.

To test the applicability of the EVS' wide dynamic range which enables the detection of subtle differences in the low luminance range, the EVS system was operated in total darkness in the deep sea at Suruga Bay (Figure S4C). For in situ observations in the deep‐sea environment, the EVS camera system with a Variable Focus Iris Lens (C‐Mount +1 mm Spacer Ring, Focal length: 4–12 mm, Aperture setting: F16–F22) was contained in a 35 MPa‐tolerant pressure housing made with aluminium alloy (Japan Industrial Standard, A7075) coated by TUFRAM^(R)^ with a 100 mm diameter viewport of 50 mm thick transparent acrylic cone (see section 4.2, Figure S5). To constrain the depth of view field and exclude distant objects that will be out of focus, a black screen was placed at 20 or 40 mm distance from the external surface of the acrylic window (Figure S5). Eight infrared LED lights (850 nm wavelength) with a total electric power of 1 W (Model number: OPTOSUPPLY OSI3XNE3E1E) were configured in a circle around the objective lens in the pressure housing (Figure S5) to provide illumination in the lightless deep sea (with the exception of bioluminescence). Silicon photodiode of the EVS exhibits comparable responsiveness from visible to invisible near‐infrared light with wavelengths up to 1100 nm, while infrared light minimises disruption to biological activities. The deep‐sea EVS system was secured on the rear side of the remotely operated vehicle (ROV) *Kaiko* (with vehicle *Mk‐IV*) facing the water column, on a dive to 1236 m deep in Suruga Bay, Japan during the final research cruise of R/V *Kairei* (KR21‐17) on January 24, 2022 (Figures S4 and S5, Kawagucci et al., 2020). The altitude of the EVS from the seafloor was approximately 1 m, where the water temperature was 3°C. Recording was conducted with infrared LEDs equipped in the pressure‐tolerant housing (Figure S5), in addition to the front‐facing LEDs for the ROV's main video cameras. The total recording time of the EVS was 3 h, from 10:00 to 13:00 JST.

### 2.2 Software

#### 2.2.1 evsCluster: Data processing software for particle identification

We newly developed a piece of software, named evsCluster, for particle identification by analysing the raw data generated through EVS recording. The entire code of evsCluster has been made publicly available on the GitHub repository of Sony Group Corporation (https://github.com/sony/evsCluster).

The evsCluster analyses the EVS outputs, consisting of pixel coordinates (*X*/*Y*), polarities in the change of brightness (+/−), and timestamps of *events*, asynchronously recorded from each independent pixel. Since the dataset generated by the EVS is quite different from conventional frame‐based cameras, it is difficult to use conventional image processing software libraries such as OpenCV for the analyses of EVS data. Analyses of the EVS outputs can be conducted simply based on the recorded raw data without producing any visual images, but a pseudo‐frame‐based image can be simulated by accumulating the EVS outputs within a short‐time interval for the purpose of visual confirmation by human eyes.

**FIGURE 3:**
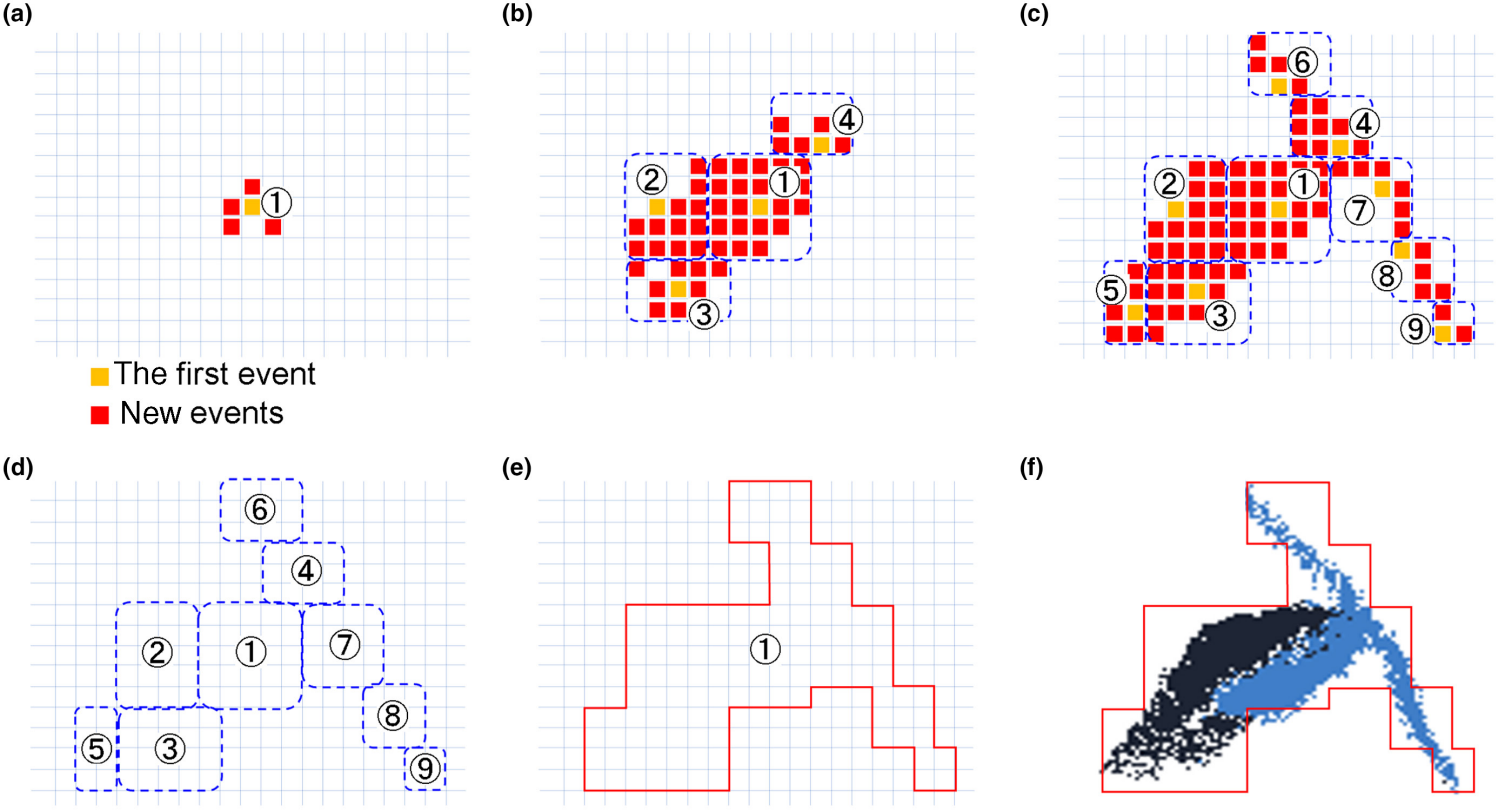
Recognition of particles and the conversion to *clusters*. (a) Labelling of the first isolated *event* (orange) with a *subcluster* ID and merging of new *events* (red) in neighbouring pixels. (b, c) Iteration of the process of (a) leads to the growth of each *subcluster*, but the growth is confined to a certain area (depicted as 5 × 5 pixels in [a–e] for illustrative purposes but actually implemented as 20 × 20 pixels in the software), allowing different *subclusters* to appear in the surrounding area. For the ease of understanding, *subcluster* 1 through 9 are depicted to appear sequentially from the centre of the particle in the figure, but in actual analysis, *subclusters* may appear and grow simultaneously from various parts of the *particle* and *subcluster* growth will stop due to the competition between neighbouring *subclusters*. (d) Only information about the size, position, and number of positive and negative *events* in each *subcluster* at each sampling time were stored in the memory. (e, f) Neighbouring *subclusters* were merged into a *cluster* whose shape generally matches the shape of the particle. The smallest *subcluster* ID is used as the *cluster* ID.

Each *event* is combined with neighbouring *events* and then output as a *cluster* by the software as follows (Figure 3). In the process of combining *events* into *clusters*, at first a small set of *events* is combined to form a *subcluster* (Figure 3a,b), and then multiple neighbouring *subclusters* are further combined to form a *cluster* (Figure 3d,e). Each *cluster* corresponds to a single particle in the real world.

In detail, simultaneous local *events* within a short period of time that are isolated from other distant *events* are recognised as a *subcluster*. Each *subcluster* is assigned to a tentative ID and is composed of *events* that occurred less than 100 ms (milli seconds) ago and all *events* older than that period were removed in order to track each *subcluster*'s movement. For each new *event*, a region of 5 × 5 pixels around the new *event* was searched to decide which *subcluster* the new *event* should be merged into (Figure 3a). Iteration of this process leads to the growth of each *subcluster*, but the growth is confined to an area of 20 × 20 pixels (Figure 3b,c). Only information related to the size, position, and number of positive and negative *events* in each *subcluster* at each sampling time was used for the following processes (Figure 3d). Naturally, a large particle exceeding the area of 20 × 20 pixels cannot fit in one *subcluster* and results in multiple neighbouring *subclusters*. Those neighbouring *subclusters* were merged into a *cluster* (Figure 3e,f). Each *cluster* is assigned to a specific ID. The combination between removing older *events/subclusters* and adding new *events/subclusters* from a *cluster* in a specific timeframe represents temporal shifts of the absolute positions of a *cluster*. The temporal shift of the *cluster* corresponds to the movement of a particle.

#### 2.2.2 Twenty‐two features used to characterise each individual particle

**FIGURE 4:**
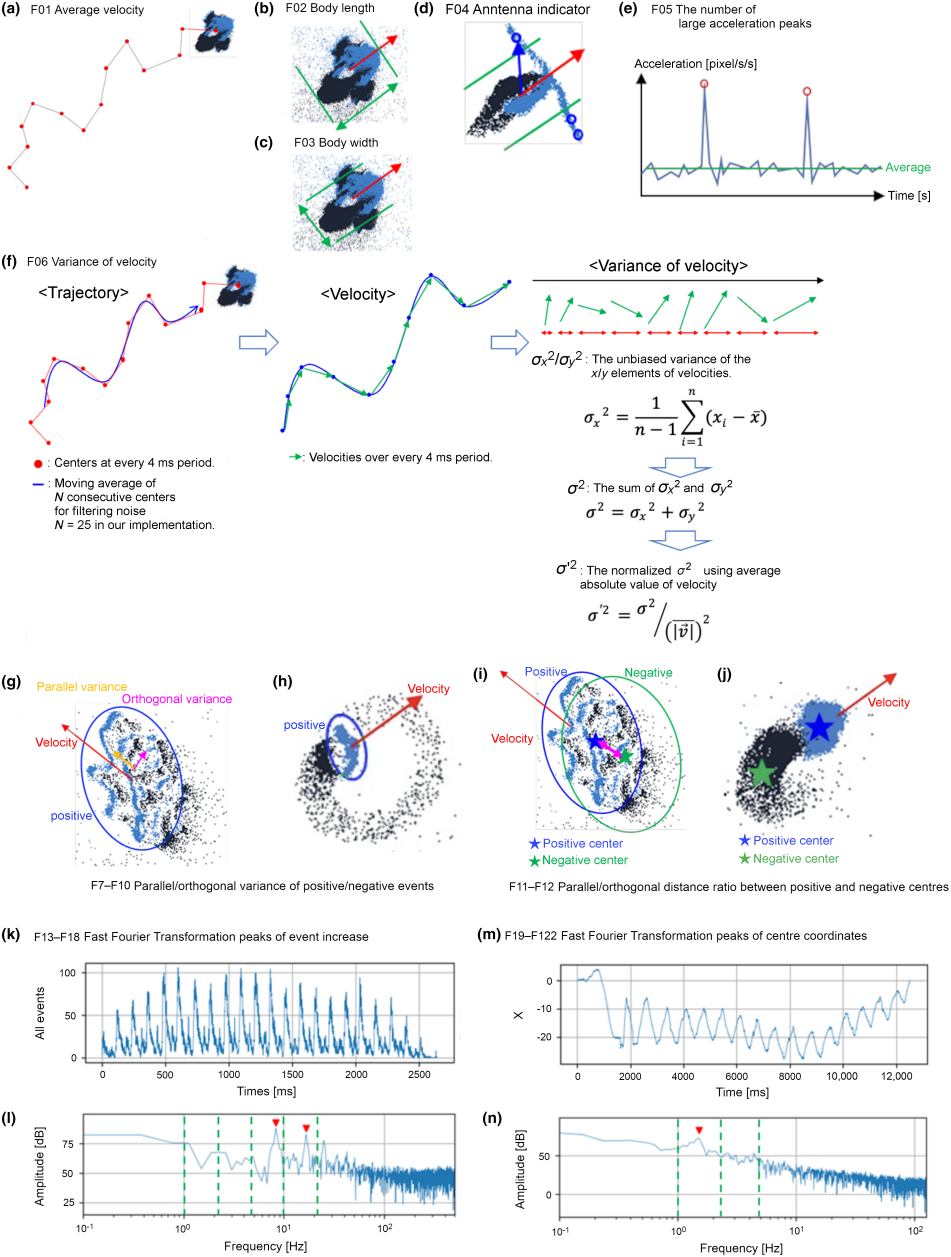
Twenty‐two features calculated to characterise *clusters* in this study. (a) F01 Average velocity: The coordinates of all *events* in a *cluster* were averaged and defined as the centre of the *cluster*. The trajectories of the centres were segmented with a fixed period of time and the velocity of the *cluster* was calculated at each segment. The velocity values were averaged across all trajectories. (b, c) F02 Body length, F03 Body width: Length and width of *cluster* were defined as the average number of pixels parallel or orthogonal to the velocity (F01), respectively. (d) F04 Antenna indicator: The inner product of the velocity (F01) and the vector from the centre to an *event* extending out of the body width was accumulated across all trajectories and divided by its length (F02). (e) F05 The number of large acceleration peaks: Acceleration was defined as the absolute value of the derivative of velocity (F01). Large acceleration peaks with values over five times greater than the average acceleration across all trajectories were counted. (f) F06 Variance of velocity: For *clusters* with an average velocity over 0.025 [pxl/ms], those with a variance of velocity higher than 0.14 were classified as *Active* or *Dynamic particles*, while the rest were classified as *Beating* or *Passive particles*. In combination with F13–F22, these *clusters* can be classified into four groups: *Active*, *Dynamic*, *Beating* and *Passive particles*. (g, h) F07–F10 Parallel/Orthogonal variance of positive/negative *events*: The average variance parallel/orthogonal to the velocity of positive *event* coordinates (blue pixels in g, h) was calculated separately and each value was divided by its length/width resulting in F07/F08, respectively. The same calculations were used on negative *events* resulting in F09/F10 (black pixels in g, h). (i, j) F11, F12 Parallel/Orthogonal distance ratio between positive and negative centres: The average distance between centres of positive and negative *events* was calculated and then divided by its length/width resulting in F11/F12, respectively. (k, l) F13–F18 FFT peaks of *event* increase: Temporal patterns of the increase of the positive, negative, and total *events* (positive and negative) of each *cluster* were analysed by Fast Fourier Transformation (FFT) with a time resolution of 1 ms. Rectangular window with the length of the entire track duration was used as the time window function. For better classification of living organisms based on the specific periodicity of particle behaviours, the entire frequency range of the power spectrum generated by FFT was divided into six segments; <1, <2.2, <4.6, <10, <22, and ≥22 Hz respectively, assigned from F13 to F18, respectively. Peaks with values greater than the background level by 15 dB in the power spectrum were counted. The peak indicates the presence of periodic movement of the *clusters* themselves. At each segment, the numbers of detected peaks were summed across all the FFT results of the positive, negative, and total *events*. (k) An example of the increase of both the positive and negative *events* at every 1 ms of an *Artemia* nauplius larva showing a saw‐toothed pattern. (l) FFT analysis of the data shown in panel (k) detected two peaks (red arrowheads) that were above the threshold. (m, n) F19–F22 FFT peaks of centre coordinates: Temporal patterns of the *x*/*y* coordinates of centres of the positive, negative, and total of positive and negative *events* of each *cluster* were analysed by FFT with a time resolution of 4 ms. A linear function was subtracted from the *x*/*y* coordinates so that the resultant *x*/*y* coordinates start and end at the coordinate of 0. Then, a rectangular window with the length of the entire track duration was used as the time window function. The entire frequency range was divided into four segments; <1, <2.2, <4.6, and ≥4.6 Hz respectively, assigned from F19 to F22. Peaks with values greater than the background level by 15 dB in the power spectrum were counted. The peak indicates the presence of periodicity in the positions of the *clusters*. At each segment, the numbers of detected peaks were summed across all the FFT results of the positive, negative, and total *events*. (m) An example of *x* coordinates of the total of positive and negative *events* in the veliger of the true limpet *Nipponacmea fuscoviridis*. (n) The result of the FFT analysis of *M* showing a peak (red arrowheads).

For our first attempt at aquatic particle observation using the EVS, we decided on the following 22 characteristic features to characterise the shape, movement, and periodicity of each *cluster* and the corresponding particle in the real world (Figure 4, Table 3). These 22 features cover the basic information of morphological and behaviour descriptions conducted in traditional zooplankton biology. As the EVS cannot directly record the shape of a particle due to its non‐frame‐based nature, the shape must be calculated from the characteristics, variances, and differences of the *events* in a *cluster* (9 characteristics out of 22). Characteristics of the *cluster* movement are also described in a similar manner (3 characteristics out of 22). To utilise EVS's advantage of high‐speed recording, millisecond‐scale periodicity in *cluster* behaviours is examined (10 characteristics out of 22). Criteria for the calculation were determined empirically without theoretical consideration and can be tuned to obtain appropriate information from the EVS output according to the research question. These 22 features are automatically calculated by evsCluster from raw data recorded by the EVS.

*F01 Average velocity*: The coordinates of all the *events* in a *cluster* were averaged, the resulting averaged coordinate was defined as the ‘centre of the *cluster*’. The trajectory of the centres was segmented with a fixed period of time (4 ms in this study) and the velocity of the *cluster* was calculated at each segment. The speed was averaged across all the trajectories (Figure 4a).
*F02‐03 Body length and width*: Length (F02) and width (F03) of each *cluster* were defined as the average number of pixels parallel/orthogonal to the velocity (F01), respectively (Figure 4b,c).
*F04 Antenna indicator*: The inner product of the velocity (F01) and the vector from the centre to an *event* extending some distance away from the body width was accumulated across all the trajectories and divided by its length (F02) (Figure 4d).
*F05 The number of large acceleration peaks*: Acceleration was defined as the absolute value of the derivative of velocity (F01). Large acceleration peaks with values over five times larger than the average acceleration across all the trajectories were counted (Figure 4e).
*F06 Variance of velocity*: For *clusters* with an average velocity of higher than 0.025 [pxl/ms], those with a variance of velocity over 0.14 were labelled as *Active* or *Dynamic particles*, while the rest were labelled as *Beating* or *Passive particles*. In combination with F13–F22 (see below), particles could be classified into four groups: *Active*, *Dynamic*, *Beating*, and *Passive* (Figure 4f, see below).
*F07–F10 Parallel/orthogonal variance of positive/negative events*: The average variance of positive *event* coordinates parallel/orthogonal to the velocity (blue pixels in Figure 4g,h) were calculated separately and each value was divided by its length/width resulting in F07/F08, respectively. Same calculations were operated on negative *events* resulting in F09/F10 (black pixels in Figure 4g,h). In general, more roughness of particle surface leads to more irregular reflection of light all over the body during motion and thus tends to exhibit a larger value of F07–F10.
*F11–F12 Parallel/orthogonal distance ratio between positive and negative centres*: The average distance between centres of positive and negative *events* was calculated and then divided by its length/width resulting in F11/F12, respectively (Figure 4i,j).
*F13–F18 Fast Fourier Transformation peaks of event increase*: Temporal patterns of the increase of the positive, negative, and the total *events* (i.e. both positive and negative) of each *cluster* were analysed by Fast Fourier Transformation (FFT) with the time resolution of 1 ms. A rectangular window with the length of the entire track duration was used as the time window function. For a better classification of plankton based on the specific periodicity of particle behaviours, the entire frequency range of the power spectrum generated by FFT was divided into six segments; <1, <2.2, <4.6, <10, <22, and ≥22 Hz, which were respectively assigned to F13–F18. At each segment, the numbers of detected peaks were summed across all FFT results of the positive, negative, and total *events*. The peaks indicate the presence of periodic movement of the particles themselves. An example of the FFT analysis of all *events* of a metanauplius larva of the brine shrimp *Artemia* is shown in (Figure 4k,l).
*F19–F22 FFT peaks of centre coordinates*: Temporal patterns of the *x*/*y* coordinates of centres of the positive, negative, and total *events* of each *cluster* were analysed by FFT with a time resolution of 4 ms. A linear function was subtracted from the *x*/*y* coordinates so that the resultant *x*/*y* coordinates start and end at the coordinate of 0. Then, a rectangular window with the length of the entire track duration was used as the time window function. The entire frequency range was divided into four segments; <1, <2.2, <4.6, and ≥4.6 Hz, assigned, respectively, to F19–F22. At each segment, the numbers of detected peaks were summed across all the FFT results of the positive, negative, and total *events*. The peaks indicate the presence of periodicity in the positions of the particles. An example of the FFT analysis of *x* coordinates of the all *events* of a veliger larva of the true limpet *N. fuscoviridis* is shown in (Figure 4m,n).

**TABLE 3:** Our proposed 22 features for characterising clusters and particles.

Name	Category	Characteristics of aquatic particles/organisms
F01	Movement	Average velocity of particle centre
F02	Shape	Length
F03	Shape	Width
F04	Shape	Antenna of organism
F05	Movement	Acceleration of movement
F06	Movement	Variance of the velocity (F01)
F07	Shape	Roughness (e.g. size/shape/distribution of bumps/ridges/bristle)
F08	Shape	Roughness (e.g. size/shape/distribution of bumps/ridges/bristle)
F09	Shape	Roughness (e.g. size/shape/distribution of bumps/ridges/bristle)
F10	Shape	Roughness (e.g. size/shape/distribution of bumps/ridges/bristle)
F11	Shape	Roughness (e.g. size/shape/distribution of bumps/ridges/bristle)
F12	Shape	Roughness (e.g. size/shape/distribution of bumps/ridges/bristle)
F13	Periodicity	Motion periodicity (<1 Hz)
F14	Periodicity	Motion periodicity (<2.2 Hz)
F15	Periodicity	Motion periodicity (<4.6 Hz)
F16	Periodicity	Motion periodicity (<10 Hz)
F17	Periodicity	Motion periodicity (<22 Hz)
F18	Periodicity	Motion periodicity (≥22 Hz)
F19	Periodicity	Movement periodicity (<1 Hz)
F20	Periodicity	Movement periodicity (<2.2 Hz)
F21	Periodicity	Movement periodicity (<4.6 Hz)
F22	Periodicity	Movement periodicity (≥4.6 Hz)

**FIGURE 5:**
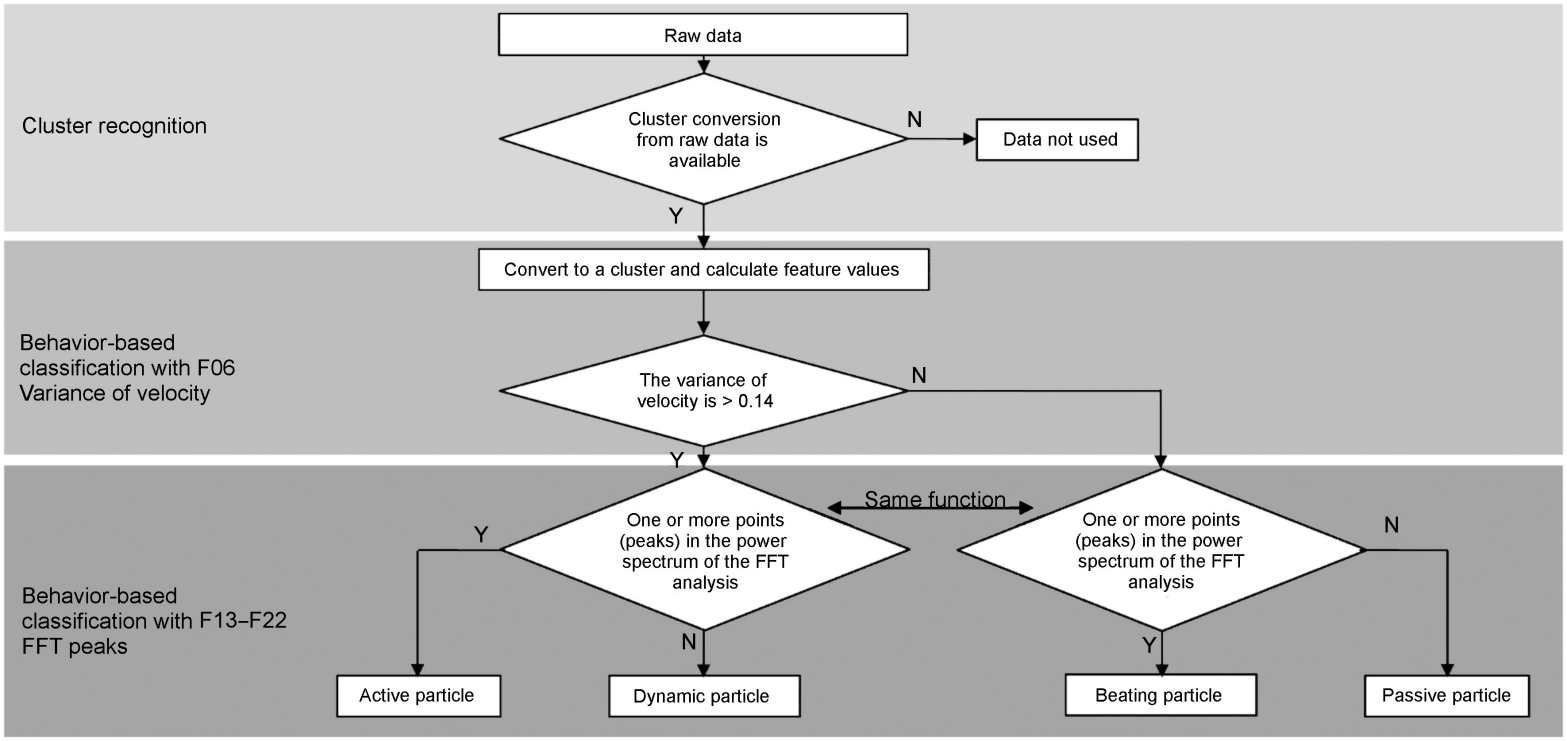
Particle classification flow. *Clusters* are mechanically classified from the raw data into the four categories of particles: *Active*, *Dynamic*, *Beating*, and *Passive* using this flow.

For *clusters* with no peak detected in the FFT analyses (i.e., values of F13–F22 were all 0), those with F6 values of 0.14 or smaller were placed in the *Passive particle* category and those with F6 values over 0.14 were categorised as *Dynamic particle*. For clusters where one or more peaks were detected in the FFT analyses (i.e., one or more of F13–F22 was ≥1), those with F6 values equal or less than 0.14 were placed in the *Beating particle* category, those with F6 values over 0.14 were considered to be in the *Active particle* category. The flow diagram of this classification of particles into the four categories is shown in Figure 5.

#### 2.2.3 Machine learning for plankton classification

To extract feature values from each species of the in vitro evaluation, we first assigned IDs to each *cluster* identified through data analysis. Then, using the frame‐based images recorded simultaneously with the EVS system, we classified each *cluster* into five categories: nauplius larva of *Parvocalanus crassirostris* (Pcra_N), copepodite and adult of *Parvocalanus crassirostris* (Pcra_C), *N. fuscoviridis* (Nfus), *Patiria pectinifera* (Ppec), and passive particles (Passive) including non‐swimming planktons and dust. After annotation of *clusters* recorded by the EVS using the frame‐based records, we selected representative individuals for each species from the entire data based on the clarity of the *cluster* shape and independence (i.e., considered to be independent when there is no collision with other particles in the 2D view).

With the EVS output from observations of the three species, the 22 features were calculated, normalised and fed into a fully‐connected three‐layer neural network (Wasserman & Schwartz, 1988) for the classification of each *cluster* into Pcra_N, Pcra_C, Nfus, Ppec, and Passive. Pcra_N and Pcra_C were distinguished for the ease of analysis and learning due to the significant difference in their movement and shapes to each other. The number of neurons in the three layers: input layer, hidden layer, and output layer, were 22, 20, and 5, respectively. Well‐known rule‐of‐thumb methods were used for determining the number of neurons in the hidden layer (Karsoliya, 2012). In preparation for the classification of a larger number of plankton species in the future, a neural network was employed to handle nonlinear separation rather than other methods such as the SVM (Support Vector Machines), which is used for linear separation between two classes. The activation function was sigmoid and the loss function was a sum of squared error. 138 *clusters* were used as teaching data, while 119 *clusters* were used as test data for validation of the analysis.

## 3 RESULTS AND DISCUSSION

### 3.1 In vitro observation of cultivated plankton

**FIGURE 6:**
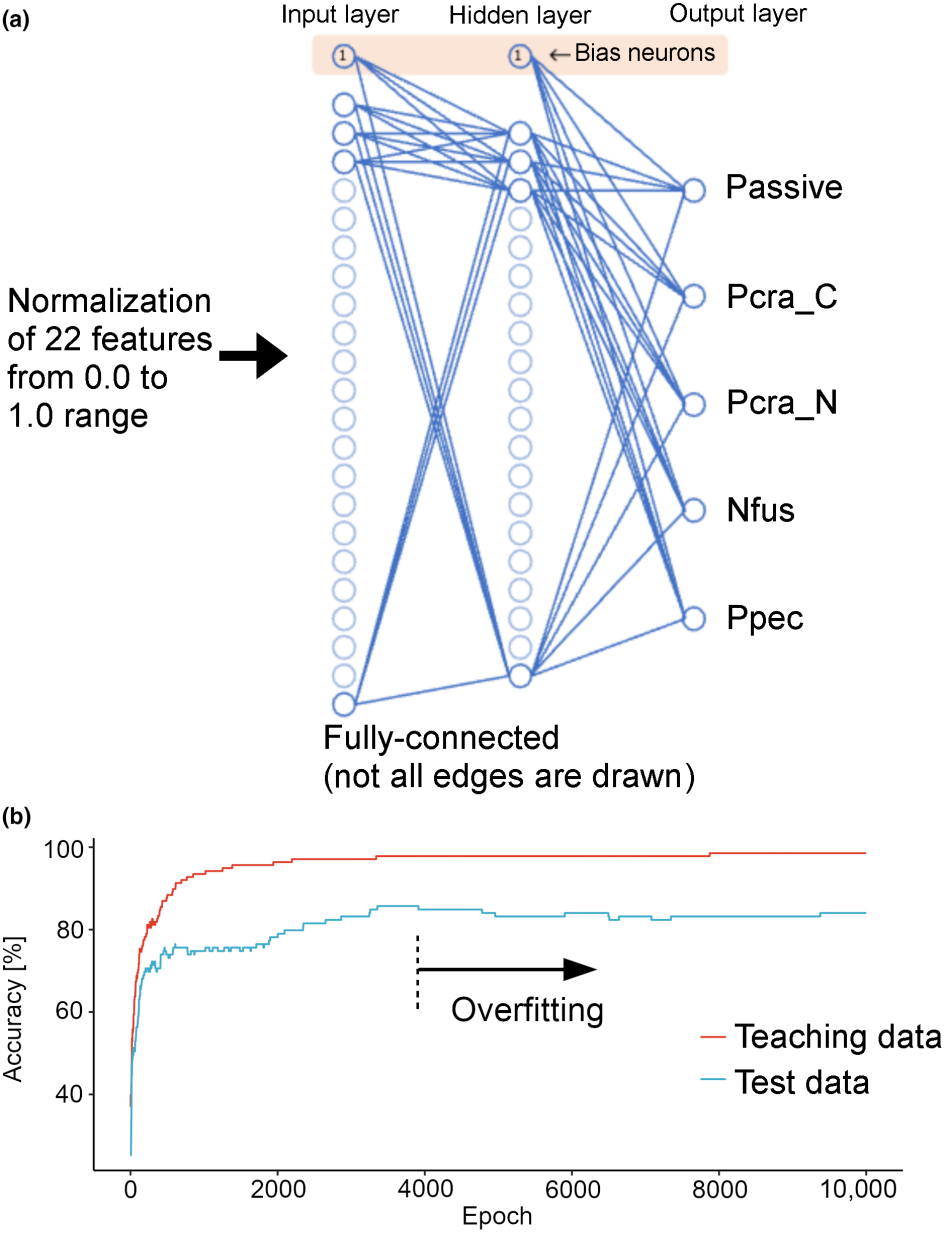
Machine learning process used for plankton classification. (a) The 22 pre‐determined characteristic features were normalised from 0.0 to 1.0 range and fed into a fully‐connected tri‐layer neural network for the classification of each *cluster* into nauplius larva of *Parvocalanus crassirostris* (Pcra_N), copepodite of *Parvocalanus crassirostris* (Pcra_C), veliger larva of *Nipponacmea fuscoviridis* (Nfus), bipinnaria larva of *Patiria pectinifera* (Ppec), and passive particle. The number of neurons of the three layers: Input layer, hidden layer, and output layer, were 22, 20, and 5, respectively. The activation function was sigmoid and the loss function was a sum of squared error. (b) Learning curve of the plankton classification. The *x*‐axis shows the number of iterations in which all the teaching data were trained (epoch), and the *y*‐axis shows the percentage of correct responses when the teaching data (red) and the test data (blue) were judged at that number of epochs. The correct response rate for the teaching data increased with the number of training sessions. When the test data was judged, the correct response rate increased at almost the same rate as the teaching data up to about 300 epochs. After that, the correct response rate of test data was lower than that of the teaching data, but the rate gradually increased to a maximum of 86%. On the other hand, after about 4000 epochs, the correct response rate either plateaued or declined, due to overfitting to the teaching data.

The 22 pre‐determined characteristic features were normalised between the range of 0.0 to 1.0 and fed into a fully‐connected tri‐layer neural network for the classification of each *cluster* (Figure 6). The correct response rate for the teaching dataset increased with the number of training sessions (Figure 6b, red). When the test dataset was run in the trained neural network, the correct response rate increased at almost the same rate as the teaching dataset up to about 300 epochs (Figure 6b, blue). After that, the correct response rate of the test dataset was lower than that of the teaching dataset, but the rate gradually increased to a maximum of 86%. This rate is slightly lower than, but comparable to, the reported value (>90%) achieved by an automated plankton image analysis using convolutional neural networks (Luo et al., 2018). On the other hand, after about 4000 epochs, the correct response rate either plateaued or declined, due to overfitting (Lawrence et al., 1997) to the teaching dataset. The high accuracy achieved by a relatively small number of teaching datasets (a total of 138) means that the EVS data contained sufficient information from which meaningful features of each species' movement and shape could be calculated. It also means that the 22 characteristic features used in this study are meaningful and sufficient for classifying a variety of living organisms and *Passive particles*. Note that our mathematical analysis of the EVS dataset recognises living organisms without any motion as *Passive particles*. The rapid movement of *clusters* and intersections between *cluster* trajectories in a 2D space often interfere with the tracking accuracy of each *cluster* when *clusters* were automatically identified by the software. The ultra‐high fps of the EVS system, however, means detailed information is available for each *cluster* until just before the intersection, allowing for the continuous tracing of a *cluster* by manual curation and therefore considerably decreasing the interference even when a large number of particles are present.

**FIGURE 7:**
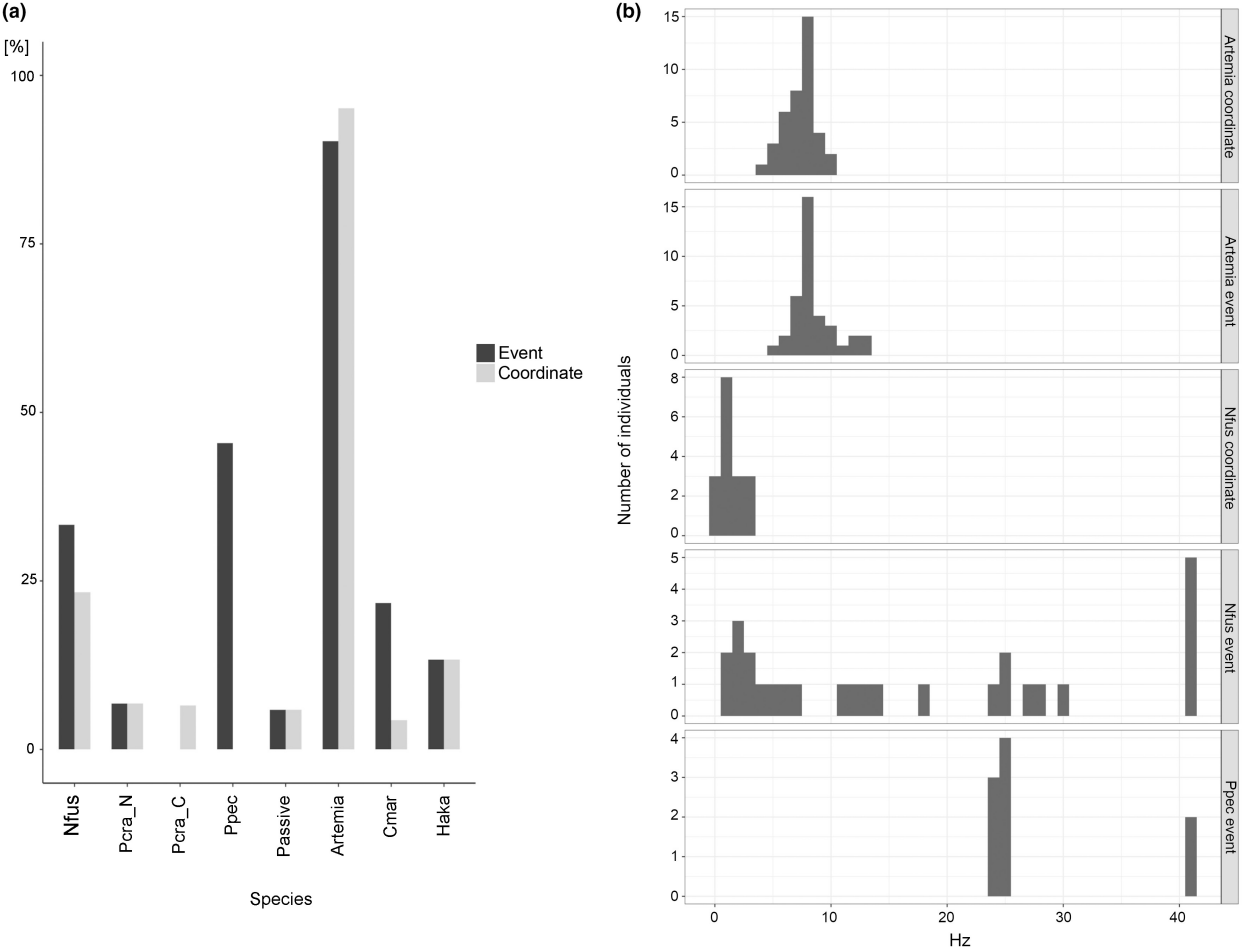
Species specificity for Fast Fourier Transformation (FFT) peaks. (a) A graph showing the percentage of individuals with FFT peaks with the increase in the number of *events* or the *x*/*y* coordinates of centres of the *cluster*. (b) Histograms of FFT peak values for each species showing that each species has a characteristic frequency.

We performed two types of frequency analyses: “event FFT peak” (F13–F18) and “coordinate FFT peak” (F19–F22). In the limpet *N. fuscoviridis*, 33.3% and 23.3% of individuals exhibited “event FFT peak” and “coordinate FFT peak”, respectively (Figure 7a, Nfus). Nauplius larvae of the copepod *Parvocalanus crassirostris* showed low percentages of both “event FFT peak” and “coordinate FFT peak”, at 6.8% (Figure 7a, Pcra_N). Copepodites and adults of *Parvocalanus crassirostris* also showed low percentages of FFT peaks, 0% in “event FFT peak” and 6.5% in “coordinate FFT peak” (Figure 7a, Pra_C). The result showing few *clusters* in this species exhibited frequencies in the EVS signals may reflect that this species does not exhibit much periodic behaviour. In the sea star *Patiria pectinifera*, while 45.5% of individuals exhibited “event FFT peaks”, no individual exhibited “coordinate FFT peaks” (Figure 7a, Ppec). Passive particles displayed a low percentage (5.9%) of both “event FFT peaks” and “coordinate FFT peaks” (Figure 7a, Passive). Metanauplius larvae of the brine shrimp *Artemia* had high percentages of individuals exhibiting both FFT peak types, 90.2% with “event FFT peaks” and 95.1% with “coordinate FFT peaks” (Figure 7a, Artemia). We also analysed the FFT peaks of the two phytoplankton species studied. *Chattonella marina* swims with two flagella, and in this species, 21.7% of individuals observed exhibited “event FFT peaks” and 4.3% had “coordinate FFT peaks” (Figure 7a, Cmar). *Heterosigma akashiwo* also swims with two flagella, and 13.3% of individuals exhibited both “event FFT peaks” and “coordinate FFT peaks” (Figure 7a, Haka). Taken together, these results indicate that plankton swimming in the water column do not always exhibit either FFT peaks, but the presence/absence of the FFT peaks provides a strong indicator for classifying actively swimming and passive particles.

We found that some plankton species tended to possess characteristic frequencies. The FFT analysis of metanauplius larvae of the brine shrimp *Artemia* showed that they exhibited “event FFT peaks” at around 7 Hz among individuals (Figure 7b, Artemia_event). The increase and decrease of *events* in this 7 Hz cycle was consistent with the anteroposterior movement of the appendages in the swimming behaviour of *Artemia* (Video 1). The 7 Hz cycle of the appendage movement resulted in the “coordinate FFT peak” being distributed around 7 Hz in this stage of larvae (Figure 7b, Artemia_coordinate). Veliger larvae of the limpet *N. fuscoviridis* tended to show “coordinate FFT peaks” at about 1.5 Hz (Figure 7b, Nfus_coordinate). This periodicity of the central coordinates reflects the spiral swimming pattern of these veliger larvae. On the other hand, there was no clear trend in the distribution of “event FFT peak” in the veliger larvae of *N. fuscoviridis* (Figure 7b, Nfus_event). Most larvae of the sea star *Patiria pectinifera* exhibited “event FFT peaks” at approximately 24.6 Hz (Figure 7b, Ppec_event); though it is unclear what behaviour this links to, it may be related to ciliary beating in the ciliary band. These results indicate that the EVS system and subsequent analyses using our software can unveil characteristics of rhythmic behaviours of organisms. The extremely high temporal resolution of the EVS enabled FFT analysis at a high‐frequency range, leading to the detection of “event FFT peaks” at over 40 Hz with both *N. fuscoviridis* and *Patiria pectinifera* (Figure 7b). This cannot be achieved with conventional cameras with only typically 50/60 Hz temporal resolution, whose frequency range would be limited to about 25/30 Hz for FFT analysis due to the sampling theorem. Some features of the current EVS, such as limited resolution and a lack of colour information (Table 1), may appear to be problematic in morphology‐based identification of a diverse range of organisms. However, the simultaneous use of the EVS and a conventional frame‐based video camera (constructed with a beam splitter as in this study) enables the complemental description of the recorded organisms by both motion and image. Such motion data recorded by the EVS and ground‐truthed by image will serve as important teaching data to further develop the potential of the EVS in the future.

**VIDEO 1:** Swimming behaviour of the brine shrimp *Artemia* sp. captured by the EVS during our in vitro observations, shown with results of the *cluster* identification.

### 3.2 In situ observation at Lake Biwa

**FIGURE 8:**
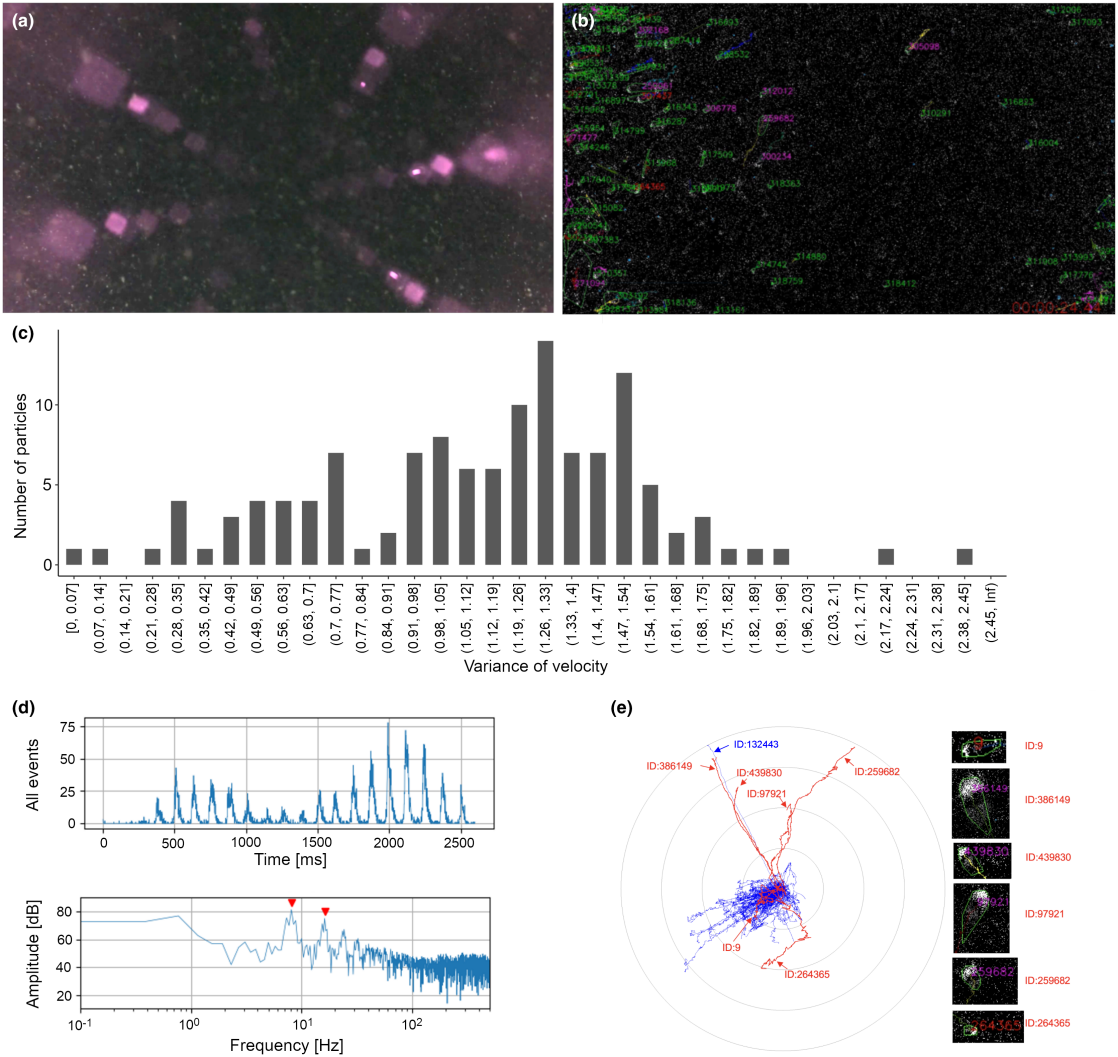
Representative results from Lake Biwa with white light. (a) A frame from video recorded by a conventional frame‐based video camera. Numerous particles were observed (whitish dots). The purple light is the light reflected on the surface of the glass sphere. (b) A snapshot of the same time as (a) in the result of analysing EVS recordings. Many *clusters* were detected. (c) A histogram of variance of velocity (F06) for a given 40 s. *X*‐axis shows the variance of velocity and *Y*‐axis shows the number of *clusters*. Most of the clusters exhibited values higher than the threshold for them to be classified as *Passive* or *Beating* particles (0.14, green dashed line). (d) The result of the FFT analysis of *cluster* ID: 386149, which was classified into *Active particle*. Upper: Increment of all *events*, lower: Power spectrum. The power spectrum shows two peaks at 8.1 Hz and 16 Hz (red arrowheads). (e) A polar chart of the *cluster* trajectories detected during the 40 s. The six clusters classified into *Active* are shown in red and the others in blue. The *Active particles* were moving in various directions, while most of the other clusters were moving to the lower left. A *cluster* (arrowhead) was classified into *Beating*, despite the almost same trajectory with an *Active particle*, ID: 386149.

Raw EVS outputs during the Lake Biwa observation were analysed to quantitatively evaluate the behaviours of particles in the natural lake water (Figure 8). During the observation period when the conventional frame‐based camera captured intensive movements of numerous particles (Figure 8a), the EVS system also detected numerous *clusters* (Figure 8b, Video 2). Although purple light from the reflection on the glass sphere surface was visible in the frame‐based image (Figure 8a), such noise caused by static illumination did not have an effect on the EVS imaging due to its nature of detecting only brightness changes. To give an example, the 122 *clusters* identified during a 40‐s recording at 11:44 JST were categorised based on the particle classification flow (Figure 5). Among the 122 *clusters*, 120 *clusters* had variances of velocity (F06) greater than the threshold of 0.14, and only two *clusters* had values less than the threshold (Figure 8c). According to the next step of the particle analysis flow (Figure 5, bottom), those 120 *clusters* with F06 over 0.14 were classified into 6 *Active* and 114 *Dynamic*. The other two *clusters* (F06 less than 0.14) were classified into one *Beating* and one *Passive*.

**VIDEO 2:** Exemplary EVS recording from our in situ observation at Lake Biwa, shown with *cluster* identification results.

All six *Active particles* exhibited periodic motion, revealed by power spectrum analysis. An *Active particle* (ID: 386149) exhibited periodic motions at the frequencies of 8.1 Hz and 16 Hz (Figure 8d). The peak at 16 Hz seems a harmonic frequency peak of the fundamental 8.1 Hz. Periodicities were also found in four of the other five *Active particles*, which were between a narrow range of 5.8 and 7.7 Hz (power spectrum not shown), while the last *Active particle* (ID: 9) had a periodicity of 1.3 Hz. The periodicity in *cluster* motion suggests that the specific periodicities correspond to characteristics of the swimming behaviour in diffferent species and their developmental stages.

Furthermore, the predominance of *Dynamic particles* compared to other particle types are unlikely to represent a collectively‐synchronised behaviour of the whole plankton community, but instead likely results from the strong water flow and the shaking of suspended particles, as well as the camera system itself shaking due to the instability of the rope‐anchored COEDO. This means that the F06 feature (variance of velocity) calculated from the dataset acquired by a floating camera system in a system impacted by water current is likely not an appropriate characteristic for distinguishing living particles from non‐life. The possible effect from the motion of the recording device on the movement of particles and animals (Iversen & Lampitt, 2020) should also be taken into consideration. Alternatively, improving the mathematical processing to eliminate components exhibiting synchronised motion, found in the great majority of *clusters*, will help to correct the effects of camera motion in *in situ* observations. This improvement on the data processing was not included in the analysis software used in the present study but should be considered in the future.

Results based on the automated classification analyses were verified by visual observation of pseudo‐frame‐based images, simulated using the 40‐s integrated subset of the EVS data that contained a total of 122 *clusters* including six *Active particles* (see above). The pseudo‐frame‐based video images (Video 2) showed that almost all the *clusters* moved in the same overall direction (to the lower left) while five *clusters* appeared to be actively moving against the direction of other *clusters* (to the upper direction). Cumulative trajectories of each *cluster* (Figure 8e) revealed that 4 of the 5 *clusters*, which showed distinct moving behaviours, were *Active particles*. This coincidence between the automated classification analyses (Figure 5) and visual observation (Video 2, Figure 8e) likely supports the validity of the evsCluster analysis. The visual observation and cumulative trajectories, however, revealed that the present automated classification sometimes mis‐classified *clusters*. A *cluster* (ID: 132443) was apparently swimming in the same direction as an *Active particle* (ID: 386149) in the video image (Video 2) but was classified into *Beating particle* due to its low variance of velocity (F06) (Figure 8e). The pseudo‐frame‐based video image also indicated that the distinct periodicity of the *Active particle* (ID: 9, see above) was a calculation artefact, due to frequent collisions/separations with neighbouring *clusters*. These two cases suggest that further mathematical optimization of *cluster* recognition, characteristic features developed to classify *clusters*, and criteria for *cluster* classification are required to improve the accuracy of automatic identification of particle types in aquatic environments.

### 3.3 In situ observation at deep‐sea in Suruga Bay

**FIGURE 9:**
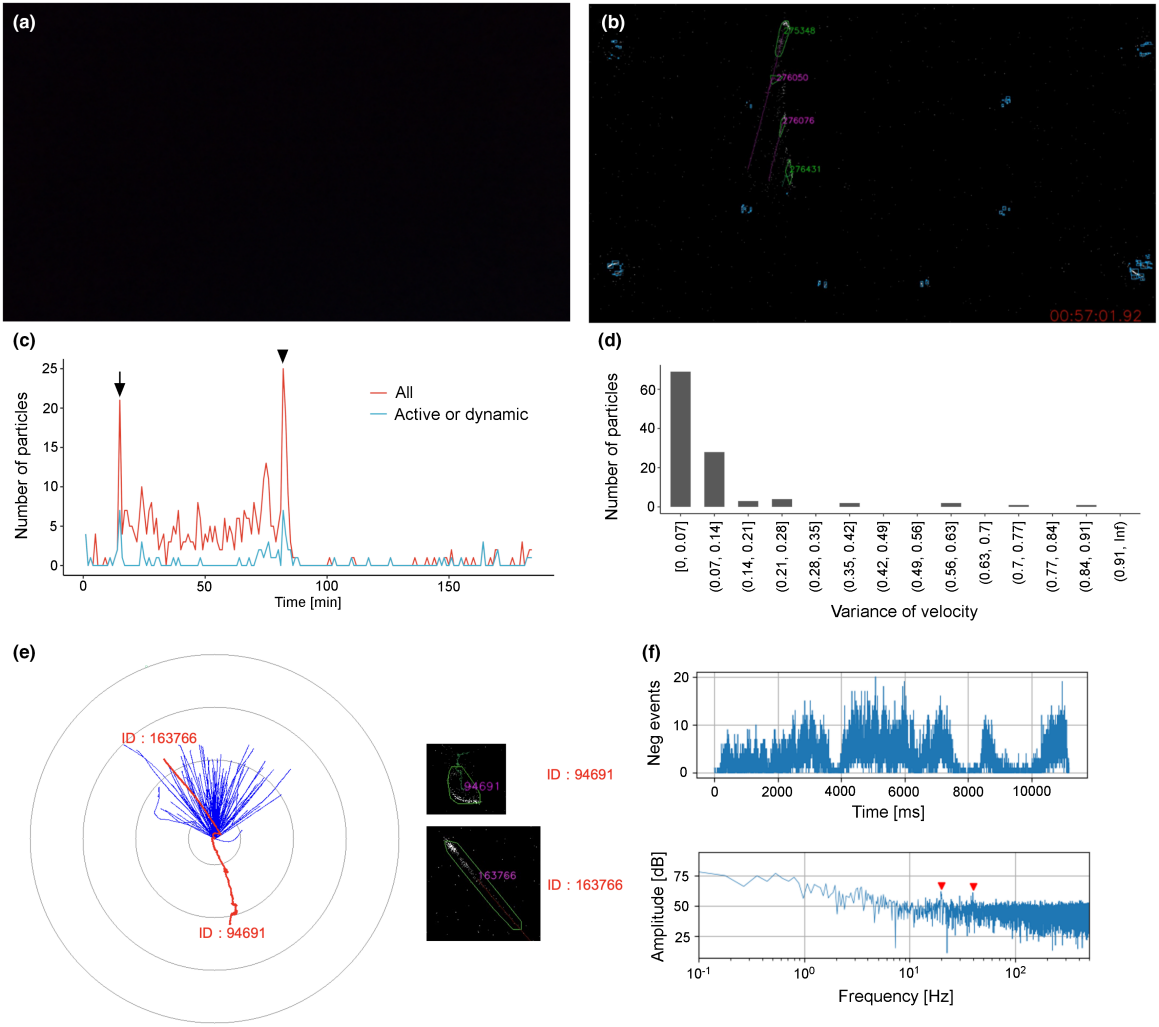
Representative results from the deep sea with infrared light. (a) A captured image from a movie recorded by the frame‐based video camera, which was unable to capture any image due to low light. (b) A snapshot of the same time as Figure 7a from the EVS showing the result of analysing its recordings. Even under very faint infrared‐light conditions, the EVS was able to detect *events* and identify *clusters*. (c) Time‐series changes in the number of *Dynamic* or *Active* (blue) and all (red) *particles* during the survey by ROV. Many *clusters* were detected at the time of the ROV arriving on the sea floor (arrow) and starting to move (arrowhead). (d) A histogram of variance of velocity (F06) for a given 20 min at the seafloor. *X*‐axis shows the variance of velocity and the *Y*‐axis shows the number of *clusters*. Almost all *clusters* were classified as *Passive* or *Beating* (threshold 0.14, green dashed line). (e) A polar chart of the *cluster* trajectories detected during the same 20 min. The two *clusters* classified into *Active particle* are shown in red and the others in blue. (f) The result of the FFT analysis of *cluster* ID: 94691, which was classified into *Active particle*. Upper: Increment of negative *events*, lower: Power spectrum. The power spectrum showing two peaks at 20 and 40 Hz (red arrowheads).

The conventional frame‐based camera (GoPro HERO8) set behind the cubic beam splitter in the deep‐sea EVS system (see Section 2.1) was unable to capture clear visuals from 100 m below the sea surface, due to insufficient light sensitivity of the sensor elements (Figure 9a). In contrast, the EVS was able to detect particles under the very faint infrared LED light (Figure 9b). During the descent to the seafloor in the deep‐sea observation at Suruga Bay, the EVS captured only a few *clusters* in the deep‐sea water column. At the time of the ROV arriving on bottom at 10:20 JST (Figure 9c, arrow), the number of *clusters* identified by the EVS increased, likely due to the resuspension of sedimentary particles from seafloor disturbance by the ROV. These *clusters* were classified into not only *Passive* or *Beating*, but also *Dynamic* or *Active* ones, based on the criteria mentioned above. Through the entire 180‐min of EVS recording, the number of *Dynamic* or *Active particles* was approximately 10 times fewer than *Passive* or *Beating clusters* (Figure 9d). Cumulative trajectories of each *cluster* as well as the pseudo‐frame‐based video, reconstructed from a 20‐min subset of the EVS recording (from 10:14 to 10:34), showed that almost all *clusters* moved upwards (Figure 9e, Video 3). Based on the particle classification flow (Figure 5), two *clusters* were classified into *Active particles* during those 20 min. One of the two (ID = 94,691) showed a distinct trajectory from the others. This indicates that, in addition to numerous particle resuspensions, a benthic animal swam against the flow (Figure 9e). The power spectrum of the negative *events* of this *Active particle* (ID = 94,691) exhibited periodic motions at the frequencies of 20 Hz and 40 Hz, while probably being harmonics (Figure 9f).

**VIDEO 3:** Exemplary EVS recording from our in situ deployment in the deep sea of Suruga Bay, shown with *cluster* identification results.

## 4 CONCLUDING REMARKS

Our tests of the EVS system in vitro in controlled laboratory aquaria as well as in situ in both lacustrine and deep‐sea environments showed that the system is capable of identifying and classifying the movement of particles in aquatic systems. Ultra‐high‐speed movement tracking and the wide dynamic range of the EVS system allow us to quantify high‐speed (millisecond‐order) rhythmic motions of planktons under weak infrared LED lighting, as well as the dynamics of planktons swimming among numerous passive particles in the relatively turbid lake water. The 22 characteristic features developed for the classification and analysis of the EVS output are sufficiently functional in distinguishing particles based on their behaviour, as seen through in situ observations and confirmed by laboratory experiments. The machine learning of particle classification achieved a maximum accuracy of 86%.

These results collectively suggest a great potential for the EVS in carrying out in situ observations of planktons and/or particles in aquatic ecosystems to understand their biology and to monitor their responses to climate change. The system is sufficiently compact and can be integrated with buoys or floats like ARGO (Roemmich et al., 2019) or autonomous underwater vehicles for the sensing of deep‐sea plankton (Ohman et al., 2019), followed by edge computing (i.e. data processing directly on the device) for analyses and classification. The EVS workflow performed in the present study can be applied to analyse the behaviour of microscopic organisms with a high temporal resolution, as well as cellular‐level motion analysis, such as sperm motility. Furthermore, the EVS has wider applications to other larger organism groups. For example, breakthroughs in tracking devices and satellite communication now allow for the so‐called bio‐logging approach to incorporate video data (Watanabe & Takahashi, 2013), and there are year‐long video monitorings of underwater communities (Aguzzi et al., 2019). The EVS can be added to these systems or replace conventional video cameras in order to gain a more in‐depth and automated understanding of organismal behaviour. In addition, many larger animals outside of aquatic environments also exhibit rapid motions, such as voice boxes of many birds and mammals during sound production. Recently, biomechanical studies of vocal fold vibration in laryngeal sound production and pathophysiological evaluation of human voice disorders have been carried out using high‐speed frame‐based video analyses (Schützenberger et al., 2016), but the EVS offers a more superior method for these applications due to its ultra‐high fps.

Observing natural phenomena using the EVS is a newly emerging method and will benefit greatly from future improvements, especially in the data analyses. For example, more appropriate and specific characteristic features to represent the particle behaviours and classification criteria could be developed. Three‐dimensional behaviour of the target object can also be reconstructed by combining multiple EVS systems (Hamann & Gallego, 2022). The accuracy of particle classification through machine learning can be improved by further iterations with more diverse teaching data and more suitable feature settings. These improvements will be the foci of future studies.

## AUTHOR CONTRIBUTIONS

**Susumu Takatsuka:** Conceptualization (equal); investigation (equal); project administration (equal); writing – original draft (equal). **Norio Miyamoto:** Investigation (lead); writing – original draft (equal). **Hidehito Sato:** Investigation (equal); software (lead); writing – original draft (equal). **Yoshiaki Morino:** Investigation (supporting); resources (equal); writing – original draft (equal). **Yoshihisa Kurita:** Investigation (supporting); resources (equal); writing – original draft (equal). **Akinori Yabuki:** Investigation (supporting); resources (equal); writing – original draft (equal). **Chong Chen:** Conceptualization (supporting); investigation (supporting); writing – original draft (equal); writing – review and editing (equal). **Shinsuke Kawagucci:** Conceptualization (equal); writing – original draft (equal); writing – review and editing (lead).

## CONFLICT OF INTEREST STATEMENT

Susumu Takatsuka and Hidehito Sato are employees of Sony Group Corporation and the Event‐based Vision Sensor IMX636 used in the present study was developed by Sony Semiconductor Solutions Corporation, which is a member company of Sony Group.

## Supporting information


Figure S1.
Figure S2.
Figure S3.
Figure S4.
Figure S5.
Figure S6.
Figure S7.

## Data Availability

Raw data used in this work are provided in Suppoting Information and Videos 1, 2, 3. The software used in this study is provided as evsCluster.zip and is also openly available on the GitHub repository of Sony Group Corporation https://github.com/sony/evsCluster.
