# Supplementary material for: Millisecond‐scale behaviours of plankton quantified in vitro and in situ using the Event‐based Vision Sensor
Source: Ecol Evol. 2024 Aug 27;14(8):e70150. doi: 10.1002/ece3.70150 (PMC11349820; doi:10.1002/ece3.70150)
Supplement: Supplementary file 1 — Figure S1. Figure S2. Figure S3. Figure S4. Figure S5. Figure S6. Figure S7. [file ECE3-14-e70150-s001.pdf]

Supplementary Information for:

Millisecond-scale behaviours of plankton quantified *in situ* and *in vitro* using the Event-based Vision Sensor (EVS)

Authors

Susumu Takatsuka[1,2,6,*], Norio Miyamoto[2,6], Hidehito Sato[1], Yoshiaki Morino[3], Yoshihisa Kurita[4], Akinori Yabuki[5], Chong Chen[2], Shinsuke Kawagucci[2,5,*]

Affiliations

[1] Sony Group Corporation

[2] X-STAR, Japan Agency for Marine-Earth Science and Technology (JAMSTEC), Yokosuka, Kanagawa 237-0061 JAPAN

[3] University of Tsukuba, Institute of Life and Environmental Sciences, University of Tsukuba, Tsukuba, Ibaraki 305-8572 Japan

[4] Kyushu University, Fishery Research Laboratory, Fukutsu, Fukuoka 811-3304 Japan

[5] Marine Biodiversity and Environmental Assessment Research Center (BioEnv), Research Institute for Global Change (RIGC), Japan Agency for Marine-Earth Science and Technology (JAMSTEC) , Yokosuka, Kanagawa 237-0061 JAPAN

[6] Equally contributed

***Corresponding authors:**

Susumu Takatsuka Email: Susumu.Takatsuka@sony.com

Shinsuke Kawagucci Email: kawagucci@jamstec.go.jp

Supplementary movies

Online Resource 2, Movie S1.

A pseudo-frame-based video image reconstructed from the EVS' observation of the swimming behavior of the metanauplius larvae of *Artemia* sp.

Online Resource 3, Movie S2.

A pseudo-frame-based video reconstructed from the EVS observation in Lake Biwa.

Online Resource 4, Movie S3.

A pseudo-frame-based video reconstructed from the EVS observation in deep-sea

Online Resource 5, evsCluster.zip.

An original code set of a evsCluster software.

Supplementary Figures

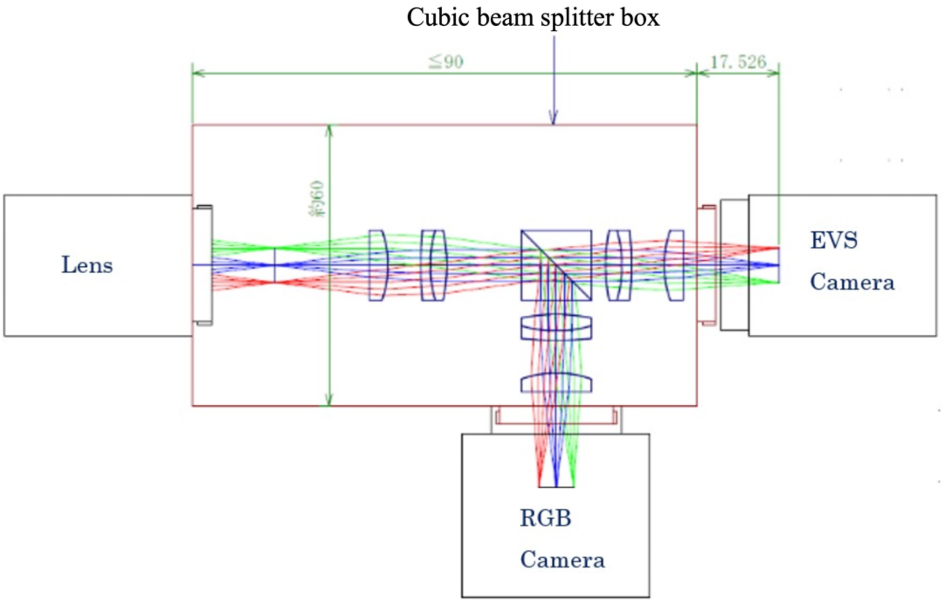

Supplementary Fig. S1. The optical axis diagram of the cubic beam splitter box (ELIOTEC CORP.)

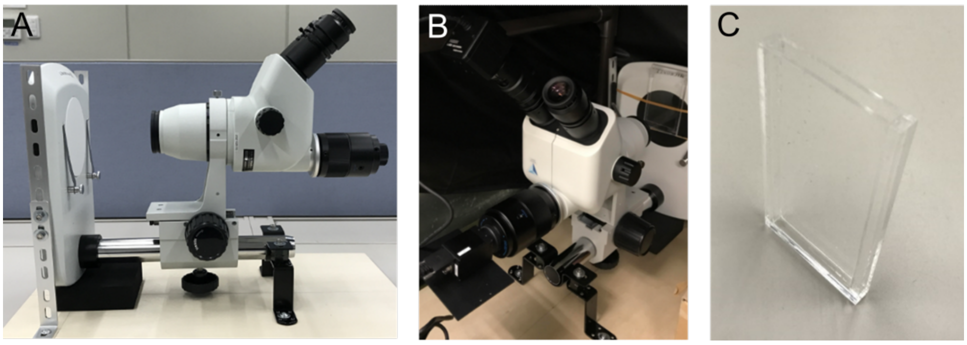

Supplementary Fig. S2. Imaging method in the *in vitro* laboratory observations. A. A stereo microscope tilted 90 degrees and fixed. **B.** An EVS camera and a conventional frame-based camera (HOZAN USB Camera L-835) connected to the microscope. **C.** The narrow aquarium used for plankton observation.

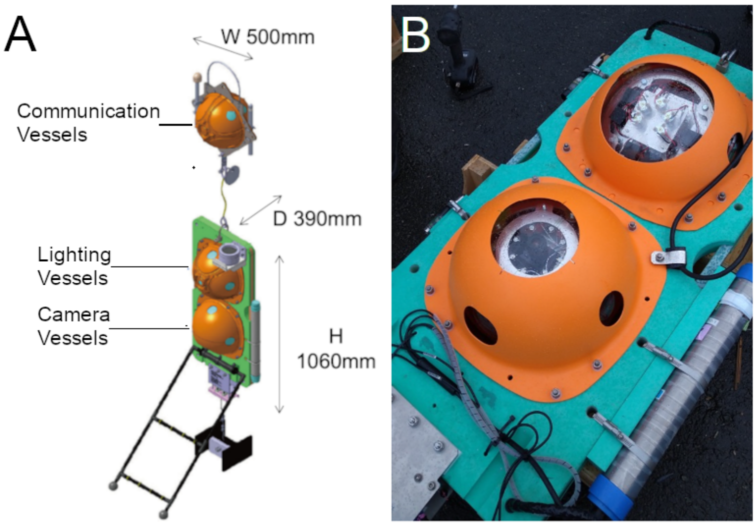

Supplementary Fig. S3. The COEDO in-situ observation system. A. A schematic image of the COEDO system. **B.** A photograph of the light and camera spheres.

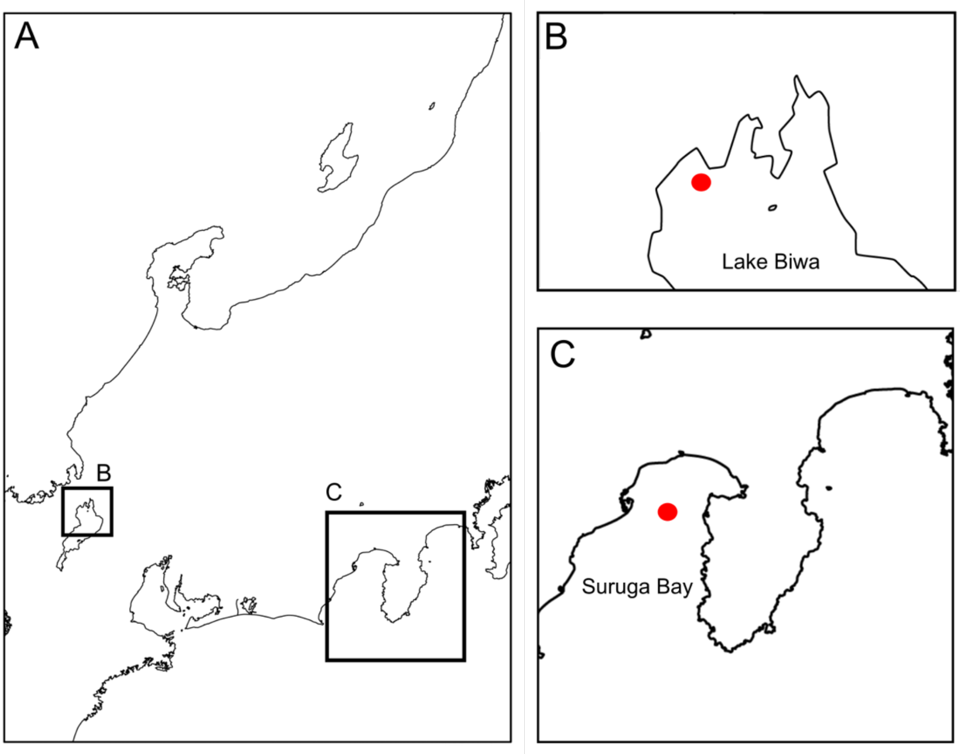

Supplementary Fig. S4. Maps showing the study sites. A. A map of central Japan showing the two research sites B (Lake Biwa) and C (Suruga Bay and Sagami Bay). **B.** A red dot showing the site where the COEDO system was deployed. **C. A** red dot showing the dive sites of ROV *Kaiko*.

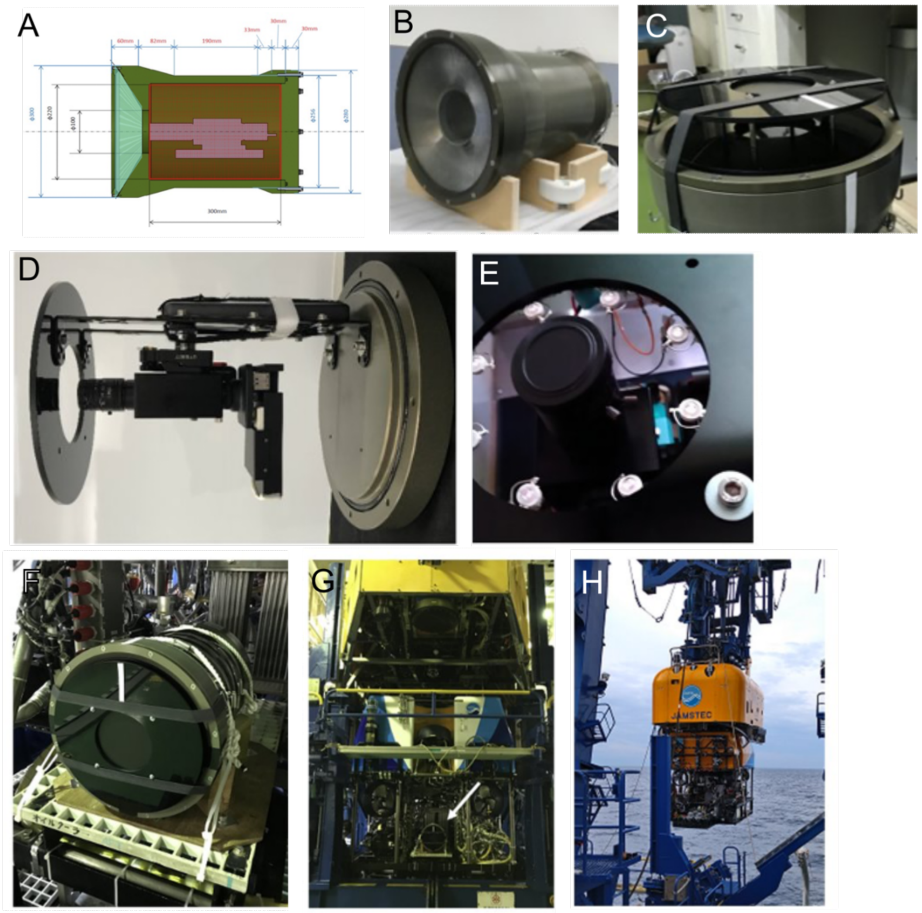

Supplementary Fig. S5. A 3,500m-class underwater camera housing used for deep-sea *in situ* observation. A. A schematic diagram of the camera housing showing that the window of the housing has a 100 mm diameter, shielded by a 50 mm thick transparent acrylic plate (in the shape of a cone). **B.** A photograph of the camera housing. **C.** A close-up view of the window showing the shielding wall to limit the depth of view. **D.** A photograph showing how the EVS prototype camera is fixed in the housing. **E.** A photograph showing 850 nm LED lights positioned around the camera lens. **F–H.** Photographs showing the EVS system being fixed at the rear of the ROV *Kaiko*.

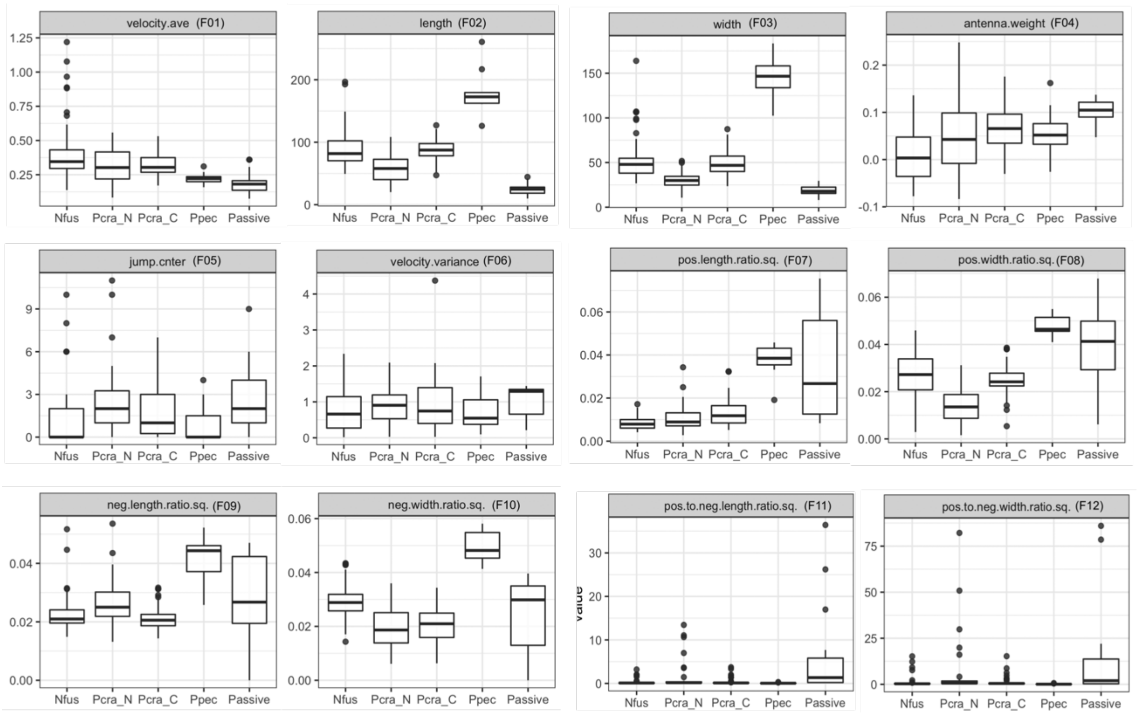

Supplementary Fig. S6. Box plots of calculated characteristic features (F01–F12) of zooplanktons and passive particles.

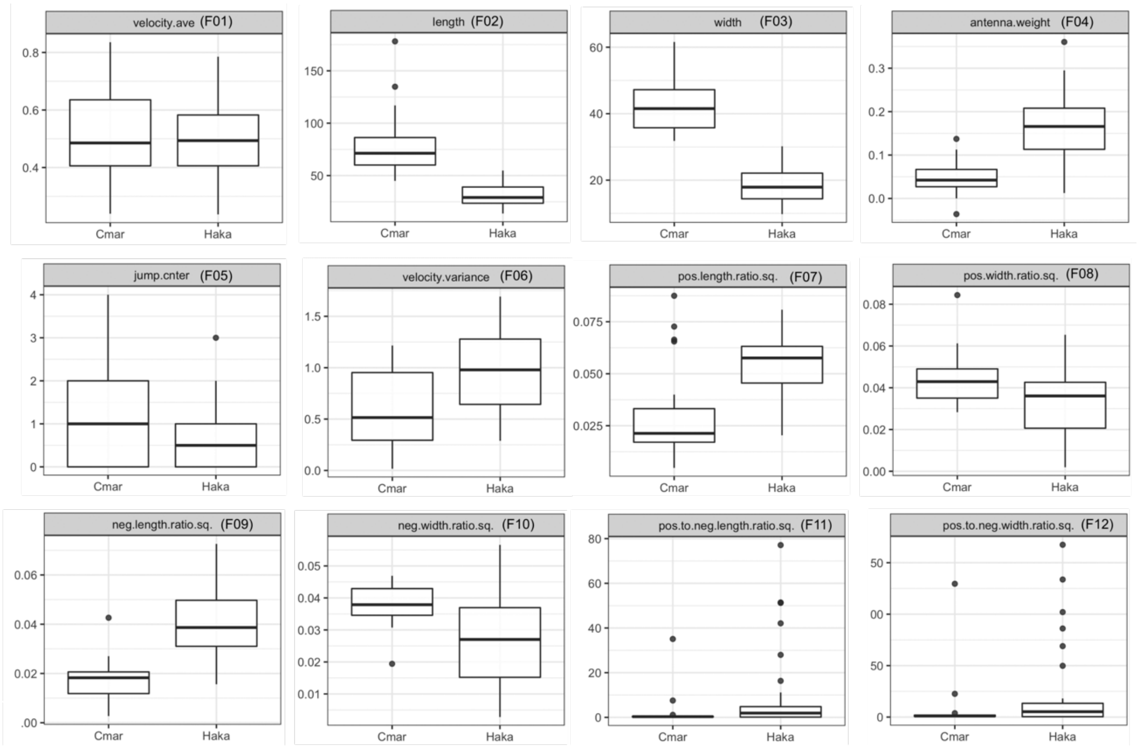

Supplementary Fig. S7. Box plots of calculated characteristic features (F01–F12) of two phytoplankton species.
